# Oridonin protects LPS-induced acute lung injury by modulating Nrf2-mediated oxidative stress and Nrf2-independent NLRP3 and NF-κB pathways

**DOI:** 10.1186/s12964-019-0366-y

**Published:** 2019-06-11

**Authors:** Huahong Yang, Hongming Lv, Haijun Li, Xinxin Ci, Liping Peng

**Affiliations:** 1 Institute of Translational Medicine, The First Hospital of Jilin University, Dongminzhu road 519, Changchun, Jilin, 130001 People’s Republic of China; 2 Department of Respiratory Medicine, The First Hospital of Jilin University, Xinmin road 71, Changchun, Jilin, 130001 People’s Republic of China

**Keywords:** Oridonin, Oxidative stress, Inflammation, Acute lung injury, Akt/Nrf2, MAPK/Nrf2, NOD-like receptor protein 3, NF-κB

## Abstract

**Background:**

Oxidative stress and the resulting inflammation are essential pathological processes in acute lung injury (ALI). Nuclear factor erythroid 2-related factor 2 (Nrf2), a vital transcriptional factor, possesses antioxidative potential and has become a primary target to treat many diseases. Oridonin (Ori), isolated from the plant Rabdosia Rrubescens, is a natural substance that possesses antioxidative and anti-inflammatory effects. Our aim was to study whether the anti-inflammatory and antioxidant effects of Ori on LPS-induced ALI were mediated by Nrf2.

**Methods:**

MTT assays, Western blotting analysis, a mouse model, and hematoxylin-eosin (H & E) staining were employed to explore the mechanisms by which Ori exerts a protective effect on LPS-induced lung injury in RAW264.7 cells and in a mouse model.

**Results:**

Our results indicated that Ori increased the expression of Nrf2 and its downstream genes (HO-1, GCLM), which was mediated by the activation of Akt and MAPK. Additionally, Ori inhibited LPS-induced activation of the pro-inflammatory pathways NLRP3 inflammasome and NF-κB pathways. These two pathways were also proven to be Nrf2-independent by the use of a Nrf2 inhibitor. In keeping with these findings, Ori alleviated LPS-induced histopathological changes, the enhanced production of myeloperoxidase and malondialdehyde, and the depleted expression of GSH and superoxide dismutase in the lung tissue of mice. Furthermore, the expression of LPS-induced NLRP3 inflammasome and NF-κB pathways was more evident in Nrf2-deficient mice but could still be reversed by Ori.

**Conclusions:**

Our results demonstrated that Ori exerted protective effects on LPS-induced ALI via Nrf2-independent anti-inflammatory and Nrf2-dependent antioxidative activities.

## Background

Oxidative stress occurs when the continuous generation of reactive oxygen species (ROS) overloads the ability of the organic antioxidative defense system and causes damage to DNA, proteins and lipids, which occurs in many human diseases such as diabetes, atherosclerosis, sepsis and acute lung injury (ALI) [1, 2]. Lipopolysaccharide (LPS), from the outer membrane of gram-negative bacteria, has been widely used to increase the production of ROS and becomes one of the most important causes of ALI [3]. The resulting neutrophil accumulation in the lungs, along with inflammatory mediators and cytokines, finally cause pulmonary edema [2, 4, 5]. Hence, finding a way to inhibit oxidative stress may be a classic target for inflammation.

Nrf2, as a vital nuclear transcriptional factor, shows strong antioxidative activity and has been widely used as a promoter to inhibit oxidative stress and the resulting inflammation [6]. As oxidative stress occurs, Nrf2 enters inside the nucleus to start the transcription of antioxidant enzymes (SOD, GSH) and antioxidant genes (HO-1, NQ-O1, GCLM), and eventually reduces oxidative damage [7, 8]. Phosphorylation of Nrf2 at serine and threonine residues by kinases such as phosphatidylinositol 3-kinase (PI3K), PKC, c-Jun N-terminal kinase (JNK) and extracellular signal-regulated protein kinase (ERK) is assumed to facilitate the release of Nrf2 and subsequent translocation [9, 10]. Previous studies suggested that AMPK activated the PI3K/Akt signaling pathway so that the phosphorylation of Akt can then activate the nuclear translocation of Nrf2 [11–13].

Thus, what are the relevant inflammatory pathways associated with Nrf2? Among the various inflammatory pathways, NOD-like receptor protein 3 (NLRP3) inflammasome and nuclear factor kappa B (NF-κB) pathways play an important role [14, 15]. NF-κB can be a sensor to start the inflammatory response under some stimuli, such as ROS, FFA, and pro-inflammatory factors. In normal conditions, NF-κB interacts with IκB to be a state of silence and have no effect on downstream genes. However, when stimulus occurs, IκB is phosphorylated and NF-κB is released and activated to control the expressions of genes and inflammatory mediators [16]. Another significant pathway, the NLRP3 inflammasome, can be activated by the assembly of NLRP3/ASC/pro-caspase 1 protein complex, resulting in the release of IL-1β [17, 18]. The release of these inflammatory factors activate many of polymorphonuclear neutrophils (PMNs), inducing “respiratory burst” and producing a large amount of reactive oxygen species (ROS) [19]. In addition, TXNIP, an inflammatory protein, also plays a considerable role. It separates with TRX-1 and binds to NLRP3 during the production of ROS [20, 21]. It is suggested that oxidative stress and the resulting inflammation have a large impact on ALI or many other diseases.

There are numerous compounds that exert antioxidative and anti-inflammatory potential through the interaction of Nrf2 and NLRP3 inflammasome and NF-κB pathways [15, 22, 23]. Oridonin, isolated from the plant Rabdosia Rrubescens, is a natural substance that has been investigated as an activator of Nrf2 and a covalent NLRP3 inhibitor [24, 25]. Furthermore, it was proven to relieve acute lung injury and vascular inflammation by blocking NF-κB pathways [26, 27]. However, there is no evidence whether the NF-κB and NLRP3 inflammasome interact with Nrf2 and the resulting protection of LPS-induced ALI. Thus in the present study, we focused on the protective effects of Ori on the oxidative stress and inflammation generated from LPS-induced acute lung injury. Additionally, we discussed whether Nrf2, activated by Ori, could mediate the inhibition of NLRP3 and NF-κB pathways.

## Materials and methods

### Reagents and chemical

Oridonin (Ori) with a purity > 98% was purchased from the National Institute for Food and Drug Control. LPS, hydrogen peroxide (H_2_O_2_), 3(4,5-dimethylthiazol-2-y1)-2,5 -diphenyltetrazolium bromide (MTT) and U0126, SB203580, LY294002, SP600125(specific inhibitors of the ERK, P38, Akt, and JNK1/2) were provided by Sigma Aldrich (St. Louis, MO). Penicillin and streptomycin, Fetal bovine serum (FBS) and Dulbecco’s modified Eagle’s medium (DMEM) were acquired from Invitrogen and Gibco (Grand Island, NY). Antibodies against Nrf2, GCLM, HO-1, P-Akt, Akt, P-JNK, JNK, P-AMPK, AMPK, P-ERK, ERK, P-P38, P38, NLRP3, CASPASE-1, ASC, IL-1β, INOS, HMGB-1, TXNIP, TRX-1, P-P65, P65, P-IκBα, IκBα, Lamin B and β-actin were supplied by Cell Signaling (Boston, MA, USA) or Abcam (Cambridge, MA, USA). The horseradish peroxidase- (HRP-) conjugated anti-rabbit or anti-mouse IgG were obtained from Proteintech (Boston, MA, USA). ToxinSensor Chromogenic LAL Endotoxin Assay Kit was purchased from GenScript (Nanjing, China). LBP and anti-LBP monoclonal antibody were procured from CLOUD-CLONE (Wuhan, China) and Santa Cruz, respectively.

### Animals

Wild-type (WT) and Nrf2^−/−^(knockout) C57BL/6 mice were purchased from Liaoning Changsheng Technology Industrial Co., LTD (Certificate SCXK20100001; Liaoning, China) or The Jackson Laboratory (Bar Harbor, ME, USA), respectively. All animals were raised under SPF-condition after feeding for several days. All studies were in accordance with the International Guiding Principles for Biomedical Research Involving Animals, which was published by the Council for the International Organizations of Medical Sciences.

### Cell culture and cell treatment

The RAW 264.7 mouse macrophage cell line, purchased from the China Cell Line Bank (Beijing China), were cultured in DMEM containing 10% FBS and incubated at 37 °C with 5% CO_2_. In all experiments, cells were allowed to acclimate for 24 h before any treatments.

### Cell viability assay

According to the manufacturer’s instructions, cell viability was evaluated by MTT assay. RAW264.7 cells were seeded in 96-well plates at the concentration of 2 × 10^4^ cells/well. Cells were added with various concentrations of Ori for 18 h before they were united with H_2_O_2_ for an hour. Then, 20 μL of MTT (5 mg/mL) was added in and the cells were incubated for another 4 h, the supernatant was replaced with DMSO to lyse the cells. Absolutely dissolved, the absorbance of MTT was measured at 570 nm.

### Intracellular ROS measurement

To detect intracellular ROS production, RAW 264.7 cells were seeded into 96-well plates (2 × 10^4^ cells/well) in DMEM for 24 h and treated with Ori (2.5, 5, 10 μM) in serum-free DMEM for 18 h. Next, the cells were incubated with DCFH-DA (50 μM) for 30 min and H_2_O_2_ (300 μM) for 10 min to trigger ROS generation. Additionally, RAW 264.7 cells were seeded into 12-well plates (4 × 10^5^ cells/well) for 24 h and then were treated with different dosages of Ori for an hour, finally exposed to H_2_O_2_ (300 μM) for 18 h. 50 μM of DCFH-DA was added for 30 min. DCF fluorescence intensities were detected by flow cytometry or a multi-detection reader at excitation and emission wavelengths of 485 and 535 nm, respectively.

### Establishment of ALI model

The treatment of animals was the same as the previous paper [22]. Briefly, the mice were randomly divided into five groups: control group, LPS group, Ori group and two LPS + Ori groups (20 and 40 mg/kg). The mice were intraperitoneal injection of Ori for 1 h, then anesthetised with diethylether, and LPS (0.5 mg/kg) was administered intranasally to induce lung injury. After LPS administration for 12 h, the animals were euthanised. Accordingly, lung tissue samples were harvested for histological evaluation and for the measurement of myeloperoxidase (MPO) activity, MDA, GSH, SOD, and NF-kB and NLRP3 pathway activation.

### Histopathological evaluation

The left lungs of the mice were immersed in normal 10% neutral buffered formalin. 5 μm sections were cut after paraffin embedding, and stained with hematoxylin and eosin (H&E) according to previous description. You can analysis the pathological changes under a light microscope.

### MPO, MDA, GSH and SOD assay in lung tissues

The right lungs were excised. The lung tissues were homogenized and fluidized in extraction buffer. MPO, MDA, GSH and SOD activity was measured using an activity kit (Nanjing Jiancheng Bioengineering Institute, China) and by measuring the change in absorbance at 460 nm using a 96-well plate reader.

### Collection of lung tissue protein and Western blot analysis

The total protein of lung tissues and cells were extracted and the protein concentrations were determined using the BCA Protein Assay Kit (Beyotime, China). After denaturation, equal amounts of protein were loaded into each well of a 10–12.5% polyacrylamide gel and subjected to sodium dodecyl sulphate polyacrylamide gel electrophoresis (SDS-PAGE). The proteins were then transferred onto polyvinylidene difluoride (PVDF) membranes, which were blocked in 5% skim milk (Sigma) at room temperature for 1 h on a shaker. Next, the membranes were incubated with primary antibodies overnight at 4 °C and subsequently washed three times for 30 min before being incubated with peroxidase conjugated secondary antibodies at room temperature for 1 h. The bound antibodies were visualised with the ECL Plus Western Blotting Detection System (GE Healthcare Buckinghamshire, UK). β-actin served as an internal protein loading control.

### LAL assay

LPS was incubated with different doses of Ori (2.5, 5, 10 μM) in the vials. Then added 100 μl LAL at 37 °C for 10 min and 100 μl reconstituted chromogenic substrate solution was added to every vials for 6 min. Stop solution and color stabilizer 2 and 3 were mixed into vials and read the absorbance at 545 nm.

### LPS-LBP-binding assay

Maxisorp 96-well plates were coated with 100 μl LPS overnight at 4 °C and blocked with BSA for 1 h. Ori was added with LBP for 1 h, and incubated with mouse anti-LBP monoclonal antibody for 1 h. Then peroxidase-conjugated goat anti-mouse IgG was added into every well and an hour later, TMB was applied for detection at 450 nm.

### Statistical analysis

All values are presented as means ± SEM. Differences between mean values of normally distributed data were analyzed using one-way ANOVA. Statistical significance was accepted when *p < 0.05 or **p < 0.01.

## Results

### Ori inhibited cytotoxicity and ROS generation in RAW 264.7 cells

Hydrogen peroxide is frequently used to induce oxidative stress in biological systems.

**Fig. 1:**
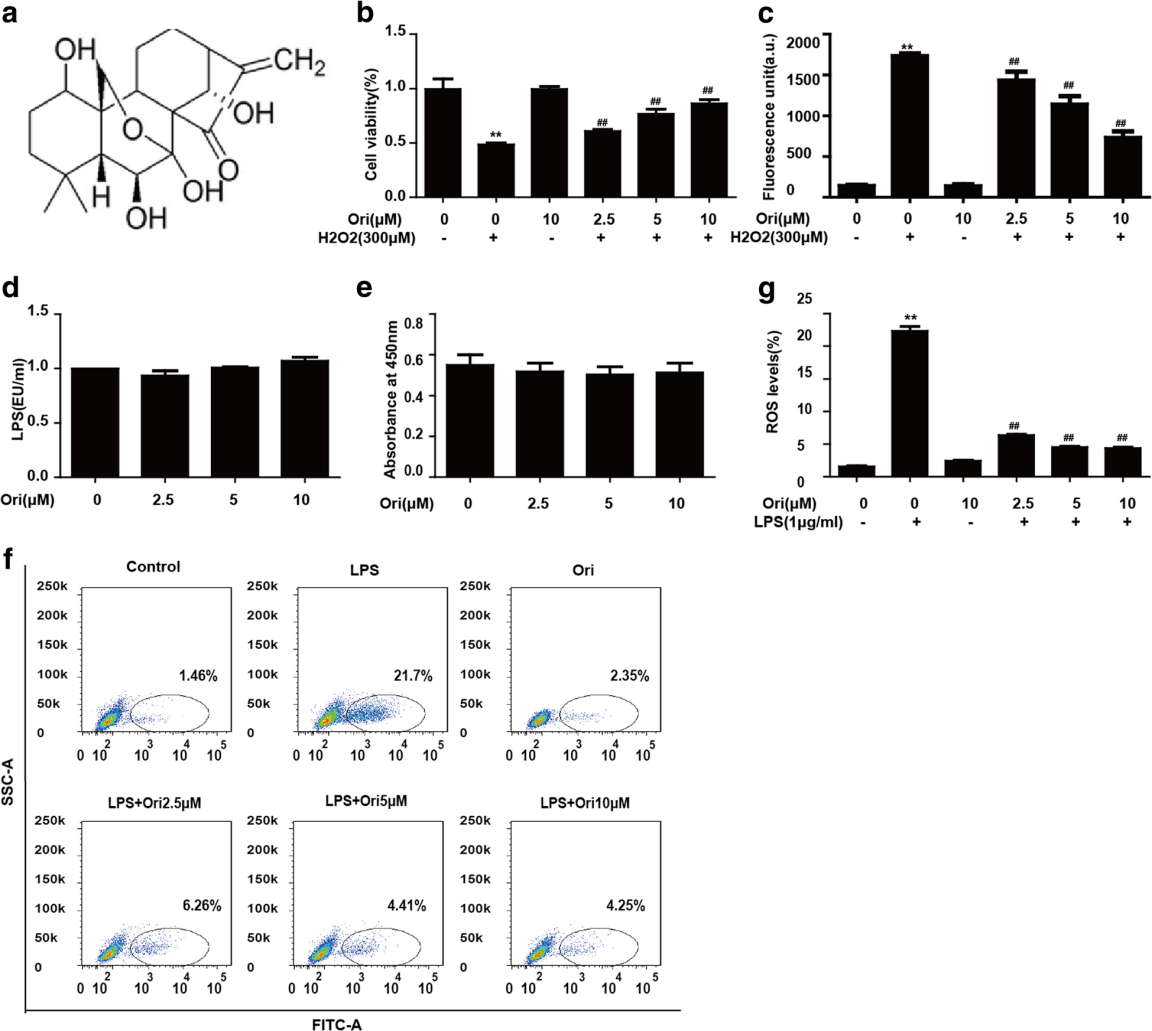
Ori inhibited cytotoxicity and ROS generation in RAW 264.7 cells. **a** The chemical structure of Oridonin (Ori). **b** Cells were exposed to Ori (2.5, 5 or 10 μM) for 1 h and then trea ted with or without hydrogen peroxide (300 μM) for another 18 h. Cell viability was determined by MTT assay. **c** Cells were treated with or without Ori for 18 h, stained with 50 μM of DCFH-DA for 30 min, and subsequently exposed to hydrogen peroxide (300 μM) for 10 min to induce ROS generation. DCF fluorescence intensities were detected by a fluorescence microscope. **d** and **e** According to the related instructions, LAL enzyme and LPS-LBP-binding assay were measured by the different absorbance. **f** and **g** Cells were exposed to Ori (2.5, 5 or 10 μM) for 1 h, treated with or without LPS (1 μg/ml) for another 18 h and stained with 50 μM of DCFH-DA for 30 min. DCF fluorescence intensities were detected by flow cytometry. All results were expressed as the means ± SEM of three independent experiments. *p < 0.05 and **p < 0.01 versus the control group, #*p* < 0.05 and ##*p* < 0.01 versus LPS group

According to previous reports [28–30], we chose 300 μM hydrogen peroxide to induce oxidative injury. Therefore, RAW 264.7 cells were pretreated with various concentrations of Ori (2.5, 5 and 10 μM) for an hour and exposed to hydrogen peroxide for 18 h. Then, cell viability and ROS generation were determined by MTT assay and fluorescence microscopy, respectively (Figs. 1b, c). Moreover, we used LPS (another stimulator) to induce ROS generation, which was determined by flow cytometry (Fig. 1f, g). Then we investigate the interaction of LPS and Ori by LAL assay and LPS-LBP-binding assay. Results showed Ori did not inhibit LPS-activated LAL enzyme and the binding of LPS with LBP (Fig. 1d, e). Our results indicated that Ori had evident cytoprotective effects in a dose-dependent manner to inhibit hydrogen peroxide- and LPS-induced cytotoxicity and ROS generation.

### Ori enhanced Nrf2 signaling pathway in a dose- and time-dependent manner in RAW 264.7 cells

**Fig. 2:**
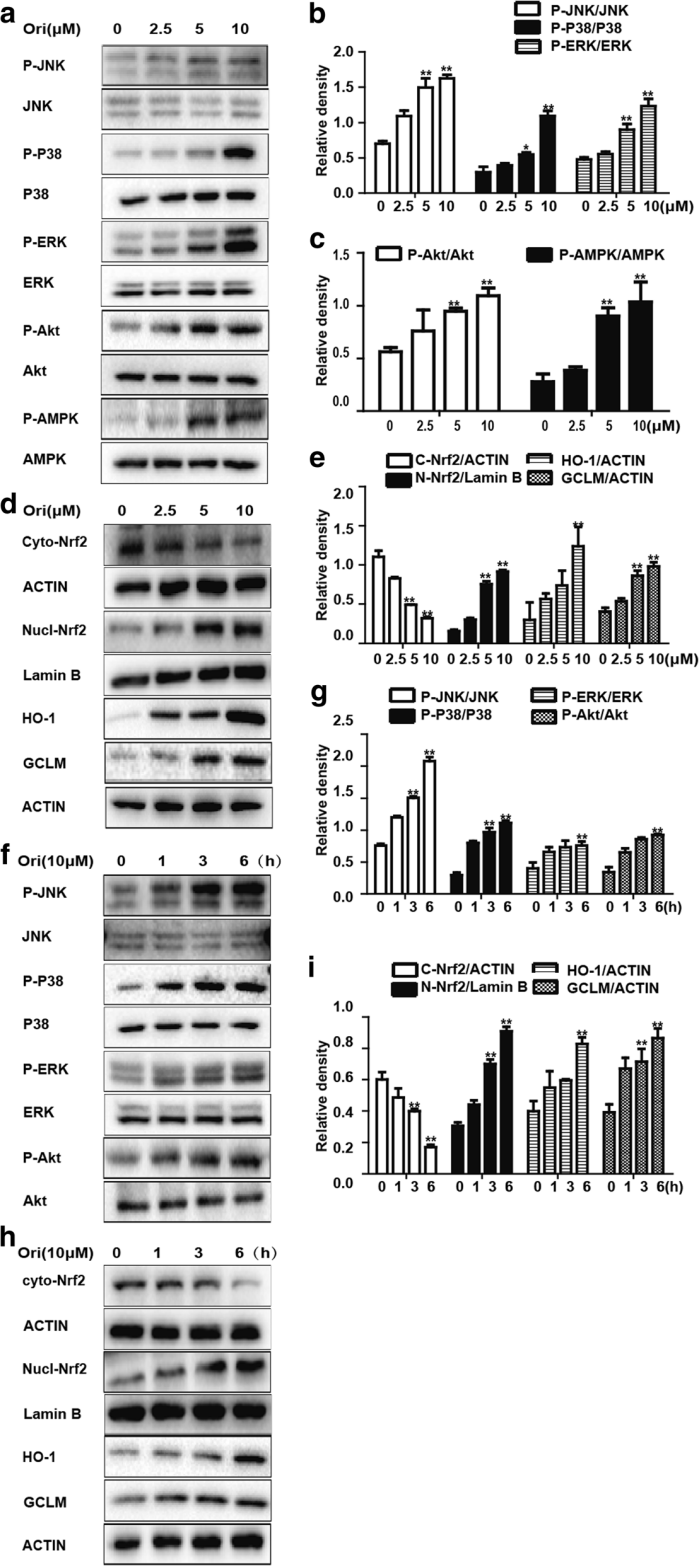
Ori enhanced Nrf2 signaling pathway in a dose- and time-dependent manner in RAW 264.7 cells. **a** and **d** RAW 264.7 cells were treated with different concentrations of Ori (2.5, 5 or 10 μM) for 6 h, or (**f** and **h**) cells were exposed to Ori (10 μM) for three time points (1, 3, 6 h). Protein expressions were measure by Western blot analysis. **b**, **c**, **e**, **g** and **i** Quantification of protein expressions were performed by densitometric analysis, and β-actin acted as an internal control for total or cytoplasmic proteins and Lamin B for nuclear proteins. All of the data shown represent the average from three independent experiments. *p < 0.05 and **p < 0.01 versus the control group

Nrf2, a nuclear transcription factor, can defend oxidative stress. It has many upstream and downstream proteins, and some were selected to investigate the antioxidative effects of Ori. RAW 264.7 cells were treated with Ori (2.5, 5 and 10 μM) for 6 h, and total protein, nuclear and cytoplasmic protein were then extracted from the cells for Western blot analysis (Figs. 2a-e). Our results showed that Ori upregulated Nrf2 expression and HO-1, GCLM expression in a dose-dependent manner. We then used 10 μM Ori for different periods of time (1 h, 3 h and 6 h), and the results indicated Ori had antioxidative effects in a time-dependent manner (Figs. 2f-i). These results suggest cells exposed to 10 μM Ori for 6 h can evidently enhance antioxidative enzyme expression.

### Ori regulated Nrf2 protein expression via Akt and MAPK activation in RAW 264.7 cells

**Fig. 3:**
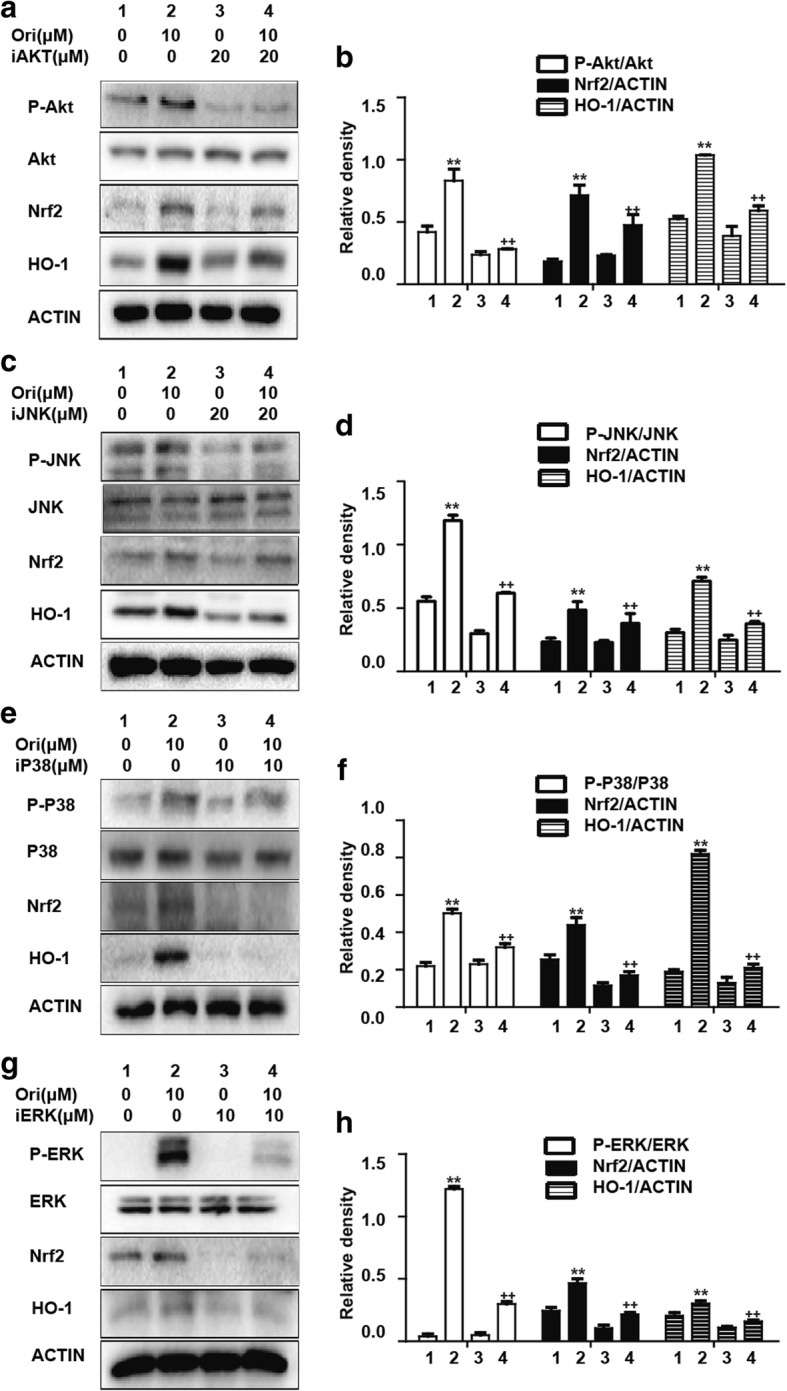
Ori regulated Nrf2 protein expression via Akt and MAPK activation in RAW 264.7 cells. **a**, **c**, **e** and **g** Cells were pretreated with or without Akt inhibitor (20 μM), JNK inhibitor (20 μM), P38 inhibitor (10 μM) and ERK inhibitor (10 μM) for 18 h, and then exposed to Ori (10 μM) for an additional 6 h. **b**, **d**, **f** and **h** Quantification of induction of Akt and MAPK phosphorylation and total Nrf2 were performed by densitometric analysis and β-actin was acted as an internal control. All results were expressed as the means ± SEM of three independent experiments. *p <0.05 and **p < 0.01 versus the control group. ^+^*p* < 0.05 and ^++^*p* < 0.01 versus Ori only group

Various signal transcription pathways, such as the AMPK and MAPK pathways, are involved in the regulation of Nrf2 nuclear transcription. In the experiments described above, we observed that Ori upregulated Akt, JNK, ERK and P38 phosphorylation (Figs. 2a-c). Moreover, we used specific inhibitors of Akt, JNK, P38 and ERK for 18 h and then added Ori for 6 h on RAW 264.7 cells. As expected, Ori-mediated Nrf2 activation was reversed by Akt and MAPK inhibitors (Figs. 3a-h). These results indicate that Ori regulated Nrf2 expression via Akt and MAPK signaling in RAW 264.7 cells.

### Ori inhibited LPS-induced inflammatory protein expressions in RAW 264.7 cells

**Fig. 4:**
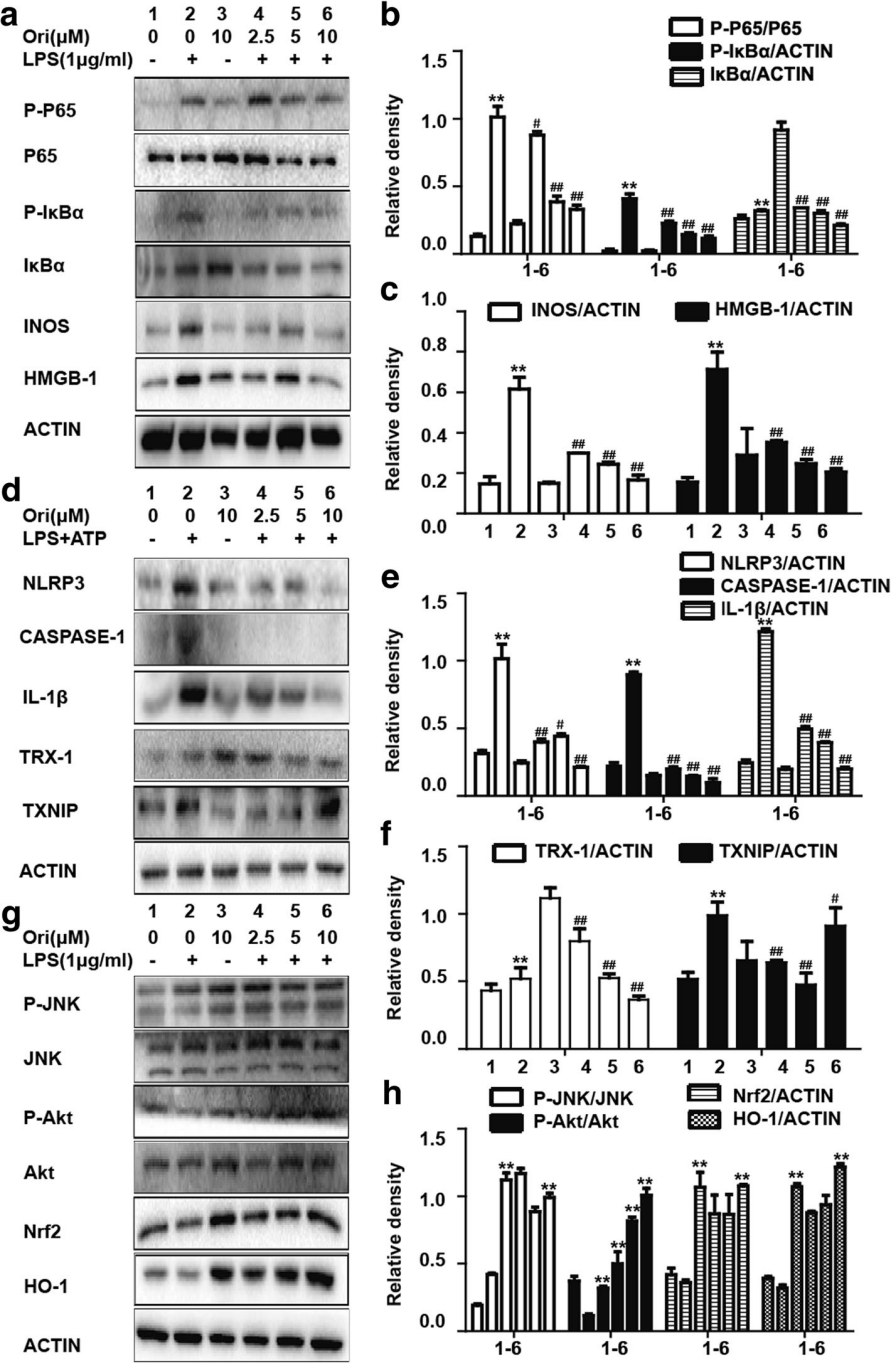
Ori inhibited LPS-induced inflammatory protein expression in RAW 264.7 cells. Cells were exposed to Ori (2.5, 5 or 10 μM) for 6 h and then treated with LPS (1 μg/ml) and ATP for 1 h and 40 min, respectively. **d** Protein expressions of NLRP3, CASPASE-1, IL-1β, TXNIP and TRX-1 were measured by Western blot analysis. Cells were exposed to Ori (2.5, 5 or 10 μM) for 1 h and then treated with LPS (1 μg/ml) for 1 h or 24 h. **a** Protein expressions of INOS, HMGB-1, P-P65, P65, IκBα and P- IκBα were measured by Western blot analysis. Cells were exposed to Ori (2.5, 5 or 10 μM) for 1 h and then treated with LPS (1 μg/ml) for 6 h. **g** Protein expressions of P-Akt, Akt, P-JNK, JNK, Nrf2 and HO-1 were measured by Western blot analysis. **b**, **c**, **e**, **f** and **h** Quantification of expressions of previous protein was performed by densitometric analysis, and β-actin acted as an internal control. All results were expressed as the means ± SEM of three independent experiments. *p < 0.05 and **p < 0.01 versus the control group, #*p* < 0.05 and ##*p* < 0.01 versus LPS group

LPS has been widely used for inflammatory models in vivo and in vitro, so it was chosen to investigate the anti-inflammatory activity of Ori. NF-κB and NLRP3 inflammasome are vital inflammatory related signals. According to our results, pretreatment of Ori inhibited LPS-induced IκBα phosphorylation and the phosphorylation of NF-κB (P65) in a dose-independent manner (Figs. 4a, b). In addition, the NLRP3 family was also inhibited by Ori (Figs. 4d, e). Apart from these two pathways, the expression of inflammatory mediators (INOS and HMGB-1) and proteins (TXNIP and TRX-1) were also repressed by Ori (Figs. 4a, c, d, f). However, Nrf2 expression was not inhibited by co-treatment of Ori and LPS (Figs. 4g, h).

### Ori exerted anti-inflammatory effects not by the regulation of antioxidative effects

**Fig. 5:**
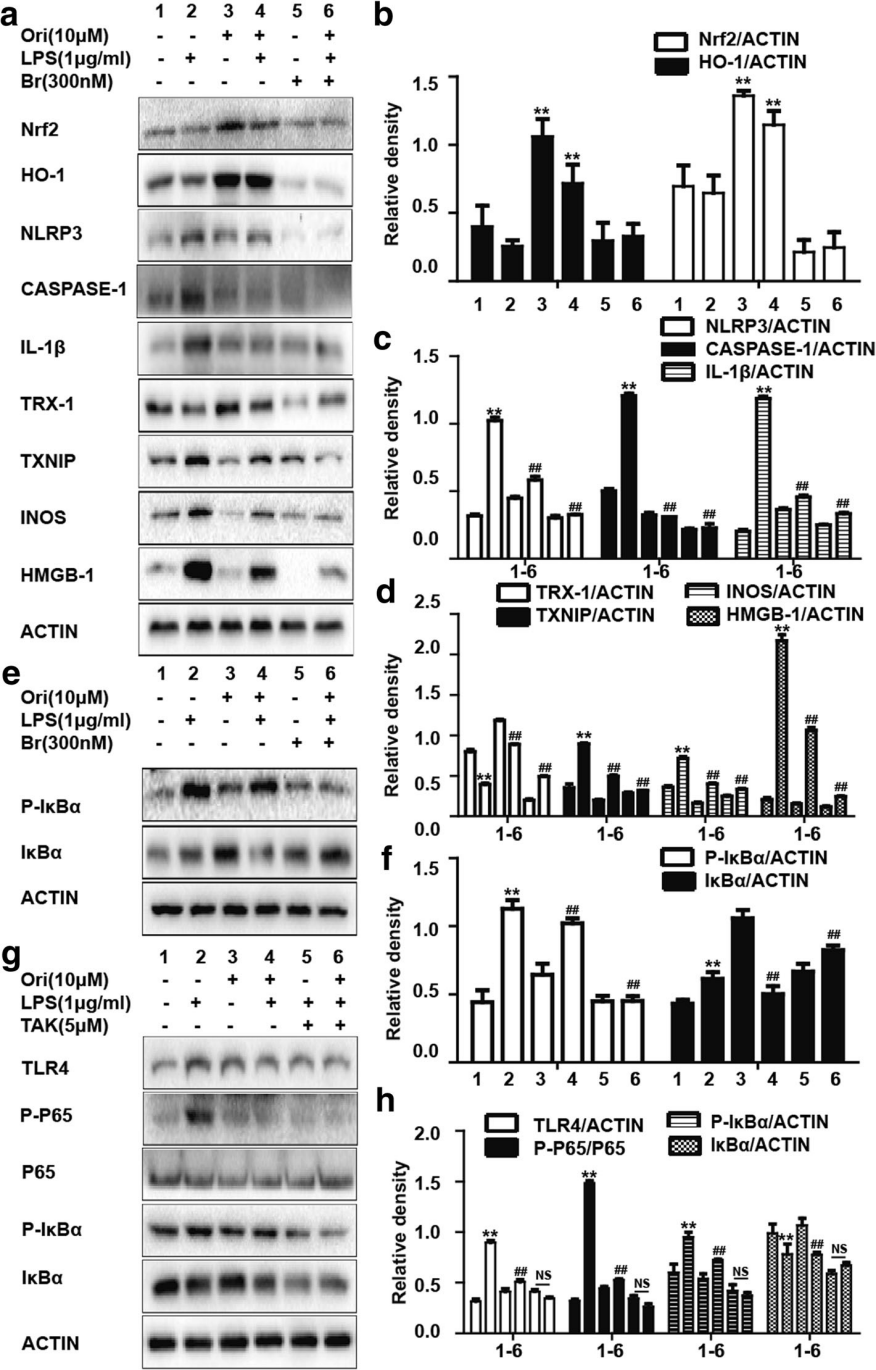
Ori exerted anti-inflammatory effects not by the regulation of antioxidative effects. After pretreatment of brusatol (300 nM) for 1 h, cells were exposed to Ori (10 μM) for 6 h and then treated with LPS (1 μg/ml) and ATP for 1 h and 40 min, respectively. **a** Protein expressions of NLRP3, CASPASE-1, IL-1β, TXNIP and TRX-1 were measured by Western blot analysis. After pretreatment of brusatol (300 nM) for 1 h, cells were exposed to Ori (10 μM) for 1 h and then treated with or without LPS (1 μg/ml) for 1 h or 24 h. **a** and **e** Protein expressions of Nrf2, HO-1, INOS, HMGB-1, P- IκBα and IκBα were measured by Western blot analysis. After pretreatment of TAK242 (5 μM) for 1 h, cells were exposed to Ori (10 μM) for 1 h and then treated with LPS (1 μg/ml) for 18 h. **g** Protein expressions of TLR4, P-P65, P65, P- IκBα and IκBα were measured by Western blot analysis. **b**, **c**, **d**, **f** and **h** Quantification of expressions of previous protein was performed by densitometric analysis, and β-actin acted as an internal control. All results were expressed as the means ± SEM of three independent experiments. *p < 0.05 and **p < 0.01 versus the control group, #*p* < 0.05 and ##*p* < 0.01 versus LPS group

Because the relationship of anti-inflammatory and antioxidative effects is the current focus of this study, we used brusatol (Nrf2 inhibitor) to see whether previous anti-inflammatory signals continued to work. We surprisingly found that the effect of Ori was not reversed by brusatol in LPS-induced RAW 264.7 cells. The phosphorylation of IκBα and NF-κB (P65) and the expression of NLRP3 inflammasome did not change (Figs. 5a-f). This may suggest that Ori exerted anti-inflammatory effects via Nrf2-independent pathways. Additionally, we added TAK242 (TLR4 inhibitor) to see whether it regulated NF-κB pathways. As our results showed, the phosphorylation of IκBα and NF-κB (P65) was declined by the use of TAK242 (Figs. 5g-h).

### Effects of Ori on histological changes and inflammatory protein expressions in LPS-induced ALI

**Fig. 6:**
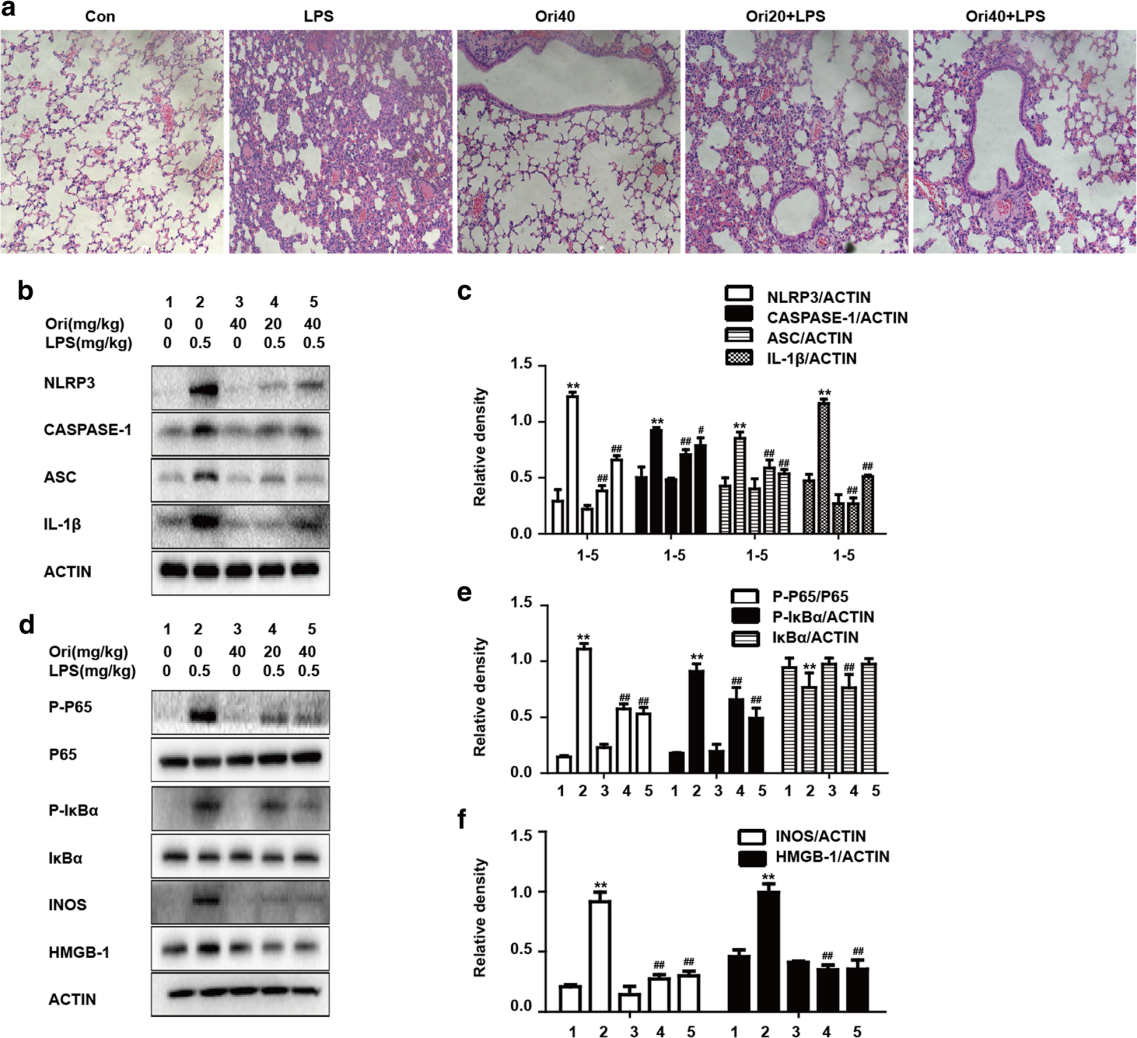
Effects of Ori on histological changes and inflammatory protein expressions in LPS-induced ALI. **a** Mice were given an intraperitoneal administration of Ori (20 and 40 mg/kg) 1 h prior to an intranasal administration of LPS. Lungs (*n* = 5) from each experimental group was processed for histological evaluation at 12 h after LPS challenge: Control, LPS group, Ori (40 mg/kg), LPS + Ori (20 mg/kg), LPS + Ori (40 mg/kg). Representative histological section of the lungs was stained by hematoxylin and eosin (H&E staining, magnification × 200). **b** and **d** Protein expressions of NLRP3, ASC, CASPASE-1, IL-1β, TXNIP, TRX-1, P-P65, P65, P-IκBα and IκBα were measured by Western blot analysis. **c**, **e** and **f** Quantification of expressions of previous protein was performed by densitometric analysis and β-actin was acted as an internal control. All results were expressed as the means ± SEM of three independent experiments. *p < 0.05 and **p < 0.01 versus the control group, #*p* < 0.05 and ##*p* < 0.01 versus LPS group

After nasal inhalation of LPS, we observed inflammatory cell infiltration and alveolar hemorrhage. However, all inflammatory infiltration was decreased by different doses of Ori (20 mg/kg and 40 mg/kg) (Fig. 6a). We then examined the effects of Ori on NF-κB and NLRP3 pathways by Western blot analysis. Consistent with vitro results, Ori decreased LPS-induced NF-κB and NLRP3 activation (Figs. 6b-f).

### Ori decreased MPO and MDA formation but increased GSH and SOD content in LPS-induced ALI

**Fig. 7:**
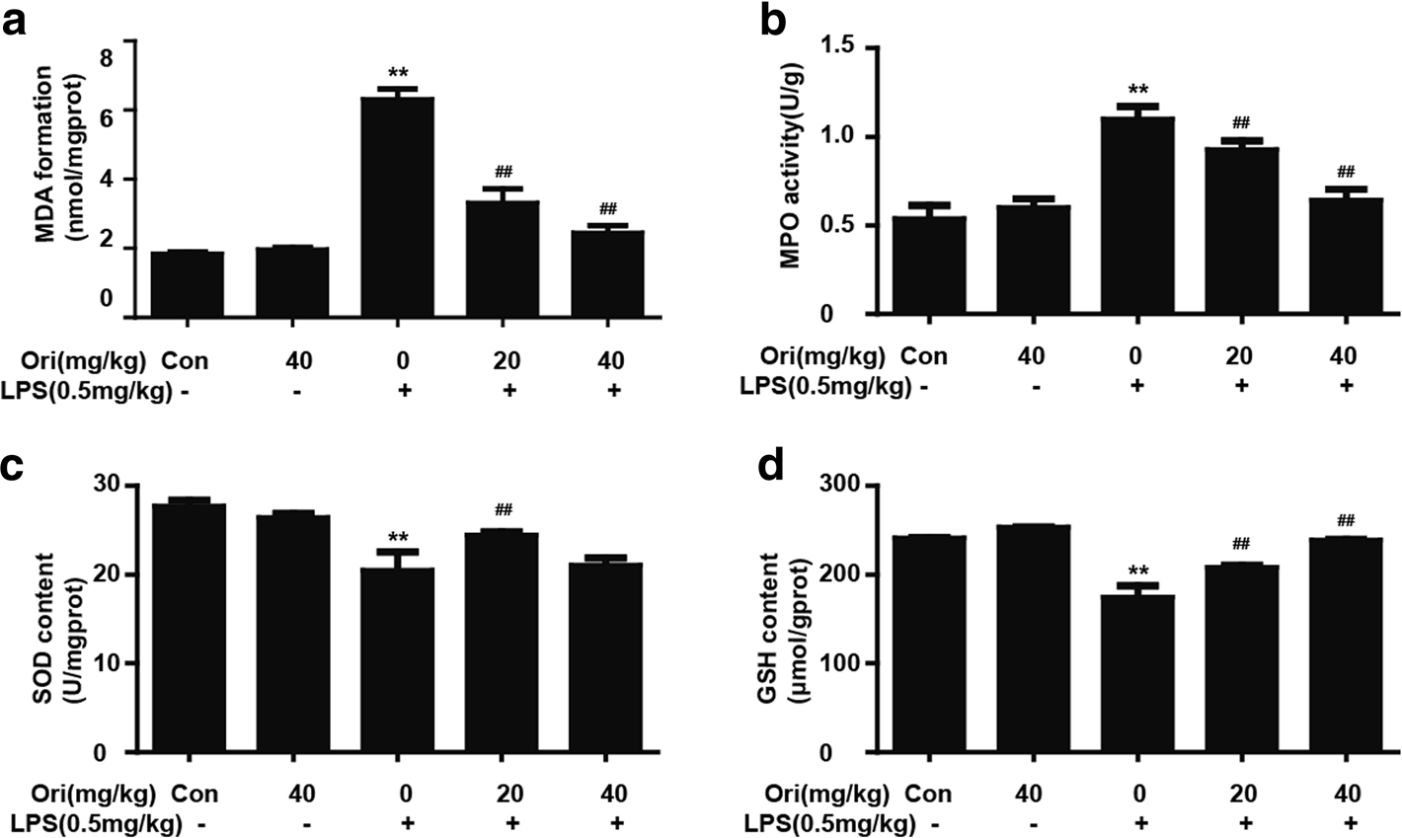
Ori decreased MPO and MDA formation but increased GSH and SOD content in LPS-induced ALI. Mice were given an intraperitoneal administration of Ori (20 and 40 mg/kg) 1 h prior to an intranasal administration of LPS. **a**, **b**, **c** and **d** MPO, MDA, GSH and SOD activity in lung tissues was determined at 12 h after LPS challenge. The values presented are the means ± SEM. (*n* = 5 in each group). *p < 0.05 and **p < 0.01 versus the control group, #*p* < 0.05 and ##*p* < 0.01 versus LPS group

Expanding on previous findings, we investigated the antioxidative effects of Ori. LPS could induce oxidative stress, which was shown by some biological indicators such as MDA, MPO, GSH and SOD content. In our research, we found that administration of LPS could increase the formation of MPO and MDA but inhibited the content of GSH and SOD, which were all reversed by pretreatment with Ori (Figs. 7a-d).

### Ori exerted anti-inflammatory effects via Nrf2-independent pathways

**Fig. 8:**
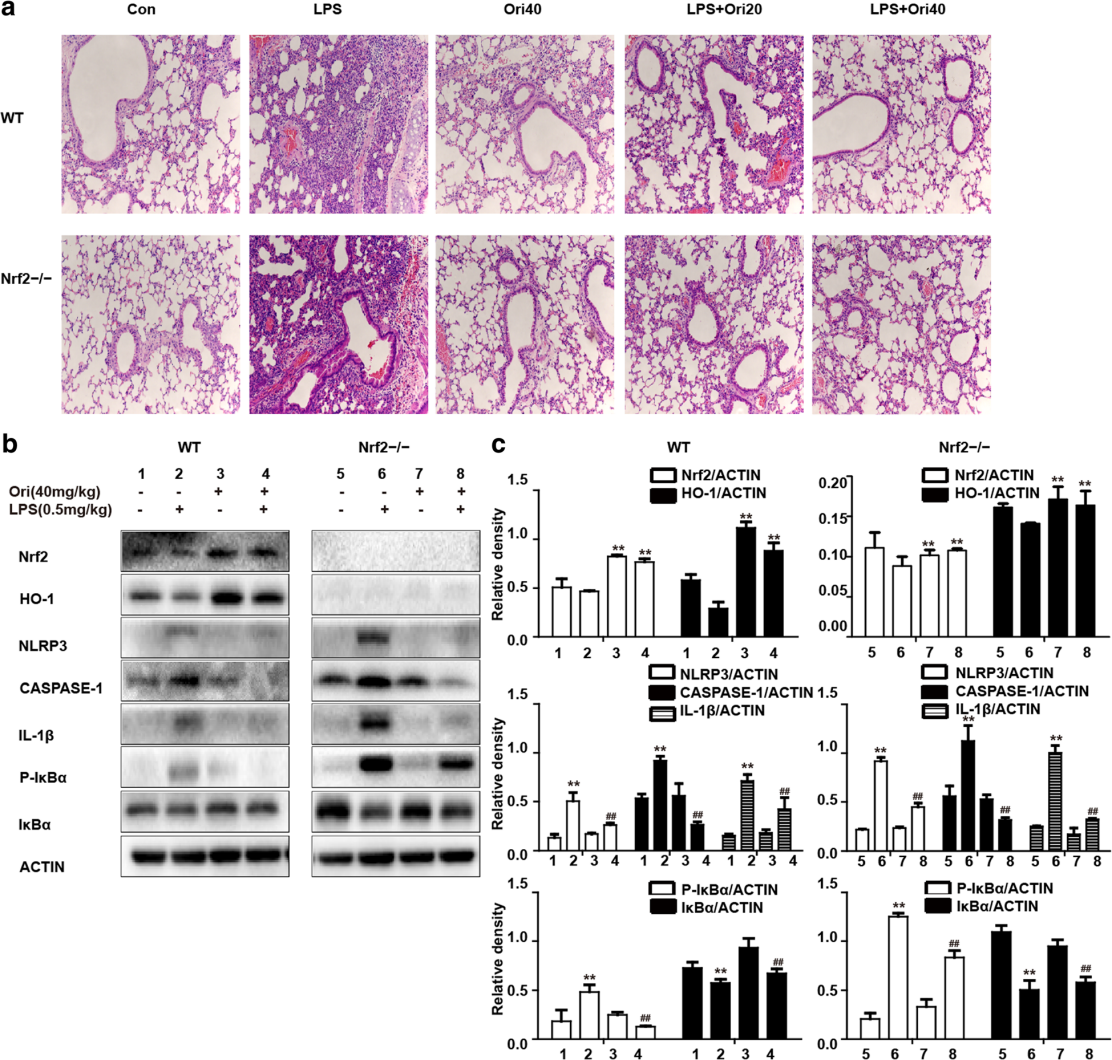
Ori exerted anti-inflammatory effects via Nrf2-independent pathways. WT and Nrf2−/− mice were given an intraperitoneal administration of Ori (20 and 40 mg/kg) 1 h prior to an intranasal administration of LPS. **a** Representative histological section of the lungs was stained by hematoxylin and eosin (H&E staining, magnification × 200). **b** Protein expressions of Nrf2, HO-1, NLRP3, ASC, CASPASE-1, IL-1β, p- IκBα and IκBα were measured by Western blot analysis. **c** Quantification of expressions of previous protein was performed by densitometric analysis, and β-actin acted as an internal control. All results were expressed as the means ± SEM of three independent experiments. *p < 0.05 and **p < 0.01 versus the control group, #*p* < 0.05 and ##*p* < 0.01 versus LPS group

To verify previous findings, we used Nrf2^−/−^mice in this experiment. In terms of histological changes, Ori alleviated LPS-induced inflammatory infiltration in WT mice and Nrf2^−/−^mice (Fig. 8a). Futhermore, the effects of Ori on NLRP3 pathway and NF-κB pathways were inhibited in LPS-induced Nrf2^−/−^mice (Figs. 8b, c). In conclusion, we could think Ori exerted anti-inflammatory activity via Nrf2-independent pathways.

## Discussion

Oxidative stress, resulting from an imbalance between the generation of oxygen radicals and the antioxidant potential in vivo, can induce inflammatory cells coming together and causing many serious diseases, including ALI/ARDS [31, 32]. To address these activities, our body possesses many defense mechanisms aimed at oxidative stress, and Nrf2 is seen as a vital component. Therefore, finding a way to inhibit the progress of oxidative stress associated with Nrf2 was the focus of this study. Oridonin (Ori), a diterpenoid isolated from *R. rubescens*, has many potential properties, including antioxidant, anti-inflammatory and anti-tumor activities. On the one hand, Ori can inhibit the production of ROS to induce the happening of apoptosis, ultimately exerts anti-tumor effects. The calculated IC50 value was 42.3 and 28.0 μM for CNE-2Z and HNE-1 cells, respectively [33]. On the other hand, Ori showed anti-ROS in normal cells and the calculated IC50 value was around 15 μM [34]. And we chose 10 μM of Ori to play the anti-ROS effects in our paper. Moreover, as an activator of Nrf2, it has a strong antioxidative property [24]. Furthermore, Ori alleviated LPS-induced inflammatory response via NF-κB pathways [26, 27]. However, the relationships between Nrf2 and inflammatory pathways have yet to be demonstrated. Our present study focused on the protective effect of Ori on ALI/ARDS and the possible mechanisms occurring on the Nrf2, NF-κB and NLRP3 pathways of this effect.

Previous reports suggested that LPS exposure could result in oxidative stress via increasing ROS formation, and so it is widely used. ROS over-accumulation can cause pulmonary edema and excessive inflammatory cell infiltration, leading to ALI [2, 4, 5]. Subsequently, internal antioxidative enzymes show up, in which SOD/GSH have extraordinary roles. SOD/GSH, efficient scavengers in possession of clearing away harmful free radicals, can prevent oxidative stress. In contrast, the over-accumulation of neutrophils and lipid peroxidation generate MPO and MDA, respectively, which act as oxidative stress pre-dominants [35]. The above four enzymes represent the trend of oxidative and antioxidative ability. In our study, Ori alleviated LPS-induced ROS production in RAW 264.7 cells and inhibited LPS-induced MPO/MDA production, GSH/SOD depletion and lung injury in mice. Additionally, it is widely accepted that the transcription factor Nrf2 effectively reduces ROS levels and competes with oxidative imbalance. Nrf2, a vital nuclear transcription factor, connects with KEAP-1 in the cytoplasm and has no effect on translocations. Once the stimuli occur, Nrf2 disassociates from KEAP-1 and binds to ARE in the nuclear. It then can promote the expressions of antioxidative genes, such as HO-1, NQ-O1, GCLC and GCLM [7, 8]. Our study showed that Ori increased the expression levels of Nrf2 and its downstream antioxidative genes such as HO-1 and GCLM. Therefore, the biological effect of Ori may be relevant to ROS clearance through the signaling of Nrf2.

Despite the role of Nrf2/KEAP-1/ARE, it is widely suggested that the PI3K/Akt and MAPK pathways play a key role in regulating Nrf2-dependent transcription [11, 12]. Therefore, we investigated whether these pathways were involved in the cytoprotection of Ori. Our results showed that Ori regulated the phosphorylation of Akt, JNK, P38 and ERK; when we used specific inhibitors of Akt and MAPK, the expression levels of Nrf2 and HO-1 were diminished, as expected. Thus, these findings suggest that Ori induced Nrf2 expression via the regulation of Akt and MAPK.

It has been determined that continuous oxidative stress can cause inflammation and occurs along with LPS-induced ALI. We know that LPS can trigger inflammatory cell infiltration and inflammatory mediator generation. Inflammatory mediators, including TNF-α, IL-1β, IL-6, INOS, COX-2 and later HMGB-1, have demonstrated important roles in inflammation-related diseases. It has been reported that Ori decreases the levels of LPS-induced pro-inflammatory cytokines (TNF-α, IL-1β, and IL-6) and alleviates lung injury via NF-κB pathways [27]. Therefore, we investigated the expressions of inflammatory genes, such as INOS and HMGB-1, and surprisingly found they were repressed by Ori in LPS-induced ALI. Many inflammatory pathways are involved, and among them, the NF-κB and NLRP3 pathways show significant effects [36]; we wanted to investigate their mechanisms. NF-κB, mainly composed of P50/P65 heterodimer, played an important role in inflammation. Once stimulated, activated NF-κB goes into the nucleus and regulates pro-inflammatory cytokines [16]. Moreover, it can promote NLRP3/ASC/pro-caspase 1 protein complex assembly, and pro-caspase 1 will be sheared into activation form, which results in the IL-1 beta and IL-18 precursors being released into the extracellular space [17, 18]. The accumulation of ROS can cause oxidative stress and increase the happening of inflammation when TXNIP is involved. TXNIP is normally located in the nucleus, but when there is an increase in reactive oxygen species, TXNIP enters the cytoplasm or the mitochondria and combines with TRX, which inhibits the antioxidative ability of TRX. It then combines with the NLRP3 inflammasome at the same time, eventually increasing oxidative stress and inflammation [20]. These pathways surely play a vital role in inflammatory responses and mediate the levels of pro-inflammatory cytokines. Thus, in our study, Ori demonstrated anti-inflammatory properties through inhibiting NF-κB and NLRP3 pathways. What’s more, LPS is recognized by LPS-binding protein (LBP), transfers to TLR4 and activates NF-κB and MAPK pathways [37]. So we investigated the effects of Ori on LPS enzyme and LPS-LBP-binding assay. Our results showed Ori did not affect the interaction with LPS, but antagonized LPS-activated cellular responses.

However, the question remains of how Nrf2 and inflammatory pathways (NF-κB and NLRP3) interact and transmit a protective effect. This has yet to be demonstrated for Ori. Hence, we added the Nrf2 inhibitor brusatol to compare the differences in LPS-induced inflammatory pathways. It showed that the treatment of brusatol and Ori had no effect on NF-κB and NLRP3 pathways. These findings were also demonstrated in Nrf2 ^−/−^ mice. In addition, LPS-induced histopathology changes were more evident than the group of WT mice and were also alleviated after Ori treatment in Nrf2 ^−/−^ mice and WT mice. Furthermore, as NF-κB upstream signals, TLR4 played an important role. So as we expected, LPS-induced the activation of NF-κB pathways were declined by the use of TAK242 (TLR4 inhibitor). These results suggest that Ori showed a protective effect in LPS-induced ALI via the Nrf2-independent NLRP3 pathway and the NF-κB pathway.

**Fig. 9:**
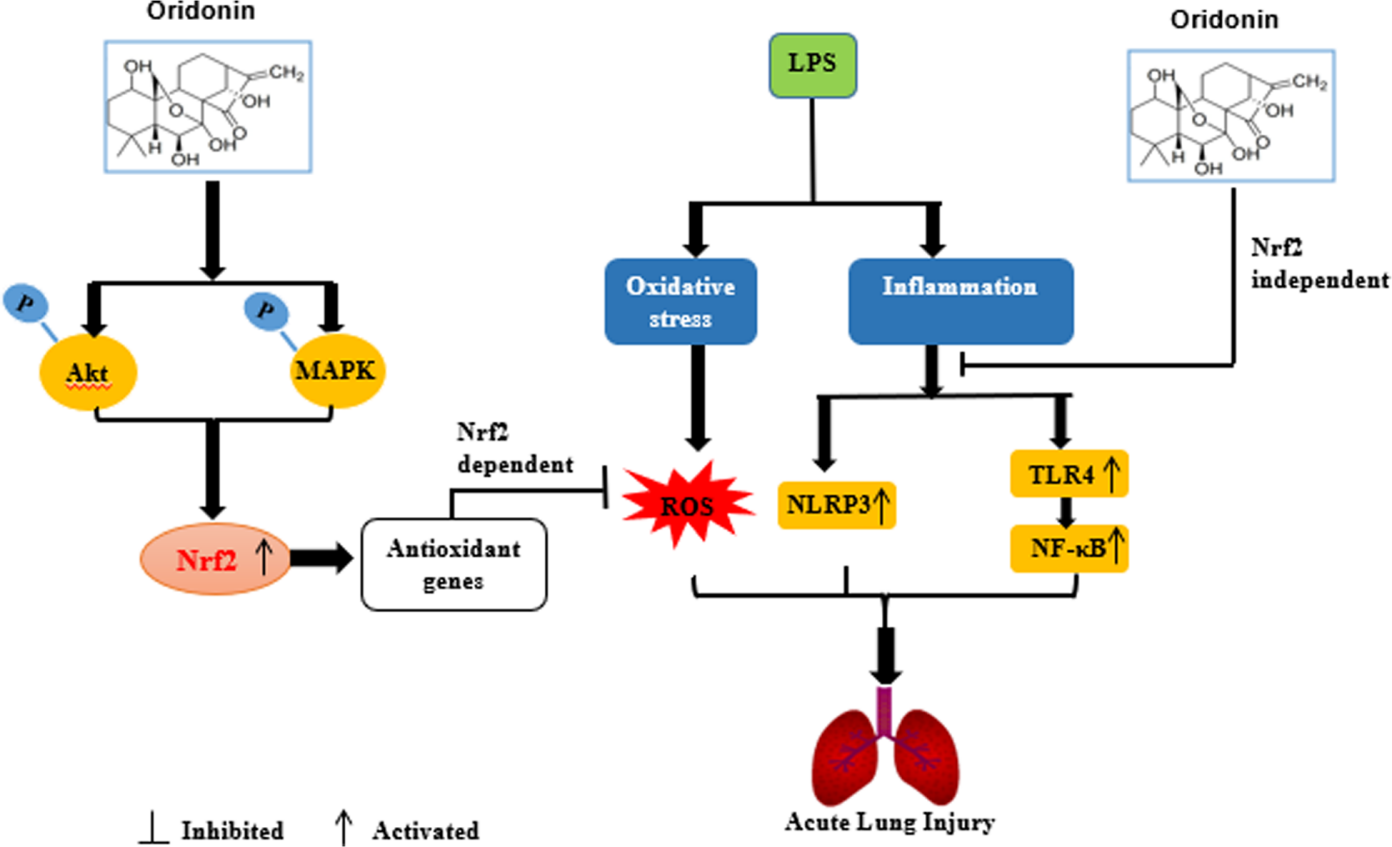
The protective effect of Ori on LPS-induced acute lung injury and the underlying mechanisms. Ori treatment significantly protected LPS-induced ALI by inhibiting oxidative stress and inflammation, which was mediated by the activation of Akt/Nrf2 and MAPK/Nrf2 and inhibition of Nrf2-independent NLRP3 and NF-κB pathways

In conclusion, as illustrated in Fig. 9, we demonstrated that Ori showed antioxidative activity in LPS-induced RAW 264.7 cells and mice via the activation of the Akt/Nrf2 and MAPK/Nrf2 signaling pathways. Additionally, two primary inflammatory pathways (NF-κB and NLRP3) were Nrf2-independent. According to the latest research, we know Oridonin forms a covalent bond with the cysteine279 of NLRP3 in NACHT domain to block the interaction between NLRP3 and NEK7, thereby inhibiting NLRP3 inflammasome assembly and activation [25]. Also, Oridonin was able to lead to accumulation of the Nrf2 protein and activation of the Nrf2-dependent cytoprotective response. And as the above, our results have demonstrated that Oridonin has superior protective effects via Nrf2-independent NLRP3 and NF-κB pathways and Nrf2-dependent antioxidative properties. Therefore, compared to other compounds, Oridonin should have better therapeutic effects, which may become a new therapy for inflammatory diseases and future studies should also address on clinical relevance of our studies.

## Conclusions

In conclusion, as shown in Fig. 9, this study suggests that Ori has a protective effect on LPS-induced lung injury. The underlying mechanisms may be closely associated with the activation of Akt/Nrf2 and MAPK/Nrf2 antioxidative pathways and inhibition of Nrf2-independent inflammatory pathways (NLRP3 and NF-κB pathways). Hence, our study gives evidence of benefit for the application of Ori in protecting against LPS-induced ALI.

## Abbreviations

Akt: Protein kinase B
ALI: Acute lung injury
AMPK: AMP-activated protein kinase
DMSO: Dimethyl sulfoxide
ERK: Extracellular signal-regulated protein kinase
GCLM: Glutamate-cysteine ligase modifier
GSH: Glutathione
GSH: Glutathione
H_2_O_2_: Hydrogen peroxide
HO-1: Heme oxygenase-1
JNK: c-jun N-terminal kinase
LBP: LPS-binding protein
LPS: Lipopolysaccharide
MAPK: Mitogen-activated protein kinase
MDA: Malondialdehyde
MPO: Myeloperoxidase
MTT: 3(4,5-dimethylthiazol-2-y1)-2,5 -diphenyltetrazolium bromide (MTT)
NF-κB: Nuclear factor kappa B
NLRP3: NOD-like receptor protein 3
Nrf2: Nuclear factor-erythroid 2-related factor 2
Ori: Oridonin
ROS: Reactive oxygen species
SOD: Superoxide dismutase
